# Salvinorin A preserves cerebral pial artery autoregulation after forebrain ischemia via the PI3K/AKT/cGMP pathway

**DOI:** 10.1590/1414-431X20176714

**Published:** 2018-03-15

**Authors:** H.P. Dong, W. Zhou, X.X. Ma, Z.Z. He, Z.H. Wang

**Affiliations:** Department of Anesthesiology, South Campus, Ren Ji Hospital, School of Medicine, Shanghai Jiaotong University, Shanghai, China

**Keywords:** Salvinorin A, Cerebral pial artery, Dilation, Forebrain ischemia, PI3K/AKT/cGMP pathway

## Abstract

This study aimed to investigate the protective effect of salvinorin A on the cerebral pial artery after forebrain ischemia and explore related mechanisms. Thirty Sprague-Dawley rats received forebrain ischemia for 10 min. The dilation responses of the cerebral pial artery to hypercapnia and hypotension were assessed in rats before and 1 h after ischemia. The ischemia reperfusion (IR) control group received DMSO (1 µL/kg) immediately after ischemia. Two different doses of salvinorin A (10 and 20 µg/kg) were administered following the onset of reperfusion. The 5th, 6th, and 7th groups received salvinorin A (20 µg/kg) and LY294002 (10 µM), L-NAME (10 μM), or norbinaltorphimine (norBIN, 1 μM) after ischemia. The levels of cGMP in the cerebrospinal fluid (CSF) were also measured. The phosphorylation of AKT (p-AKT) was measured in the cerebral cortex by western blot at 24 h post-ischemia. Cell necrosis and apoptosis were examined by hematoxylin-eosin staining (HE) and TUNEL staining, respectively. The motor function of the rats was evaluated at 1, 2, and 5 days post-ischemia. The dilation responses of the cerebral pial artery were significantly impaired after ischemia and were preserved by salvinorin A treatment. In addition, salvinorin A significantly increased the levels of cGMP and p-AKT, suppressed cell necrosis and apoptosis of the cerebral cortex and improved the motor function of the rats. These effects were abolished by LY294002, L-NAME, and norBIN. Salvinorin A preserved cerebral pial artery autoregulation in response to hypercapnia and hypotension via the PI3K/AKT/cGMP pathway.

## Introduction

Global cerebral ischemia, usually seen in clinical conditions such as cardiac arrest, drowning, or systemic hypotension during surgery (1), is associated with high mortality rates (2). Neuronal death and behavioral dysfunction caused by brain injury-induced ischemia are common in patients with stroke. Studies have shown that ischemia could change the vascular tension and reduce response of vessels, thereby leading to disruption of the blood flow around the ischemic area (3,4). The accompanying hypercapnia and hypotension could result in the dysfunction of the vessel and final cell injury in the brain. Currently, autoregulation of blood vessels is considered to play a critical role in the neuronal protection after ischemia during the hypercapnia and hypotension condition (5,6). It is necessary to maintain the autoregulation function of the blood vessel to preserve the normal blood supply and cell metabolism. However, until now, no effective therapeutic drug or strategy is available.

Salvinorin A, a bioactive substance derived from natural plants, is a highly selective and effective agonist targeting Kappa opioid receptors (KOR). Salvinorin A has an ability to pass through the blood-brain barrier (BBB) to exert its effect on brain function (7). Salvinorin A could potentially protect the brain and improve neurological outcome via BBB protection, apoptosis reduction, and inflammation inhibition (8). Compared with other KORs, salvinorin A has the advantages of having low circulation toxicity and causing no respiratory repression; therefore, it exhibits a more promising future for clinical application. Studies have shown that salvinorin A could exert vessel dilation function in the physiological condition and preserve the autoregulation of brain vessels (9,10), as well as protect against forebrain ischemia-induced brain injury (11). However, the detailed mechanisms of salvinorin A on the protection of cerebral pial artery after ischemia have not been fully explored.

The activation of the PI3K/AKT pathway has been found to have a protective effect on ischemic BBB damage (12) and induces BBB permeability after cerebral ischemia (13). The PI3K/AKT pathway is involved in the regulation of nitric oxide synthase (eNOS) and exerts its function in multiple pathological conditions (14,15). Nitric oxide (NO), generated by NOS, is considered the key factor in endothelial cell function regulation and participates in the regulation of vessel dilation and blood flow increase through elevating cGMP levels (16).

Our preliminary data have shown that salvinorin A increased cGMP levels in the cerebrospinal fluid (CSF); thus, we aimed to evaluate if the PI3K/AKT pathway is involved in the effect of salvinorin A on cGMP levels.

## Material and Methods

### Ethics statement

The study protocol (B-2016-069) was approved by the Institutional Animal Care and Use Committee of Shanghai Jiaotong University School of Medicine, Shanghai, China following generally accepted international guidelines for animal experimentation.

### Experimental animals and forebrain ischemia model

**Figure 1.**
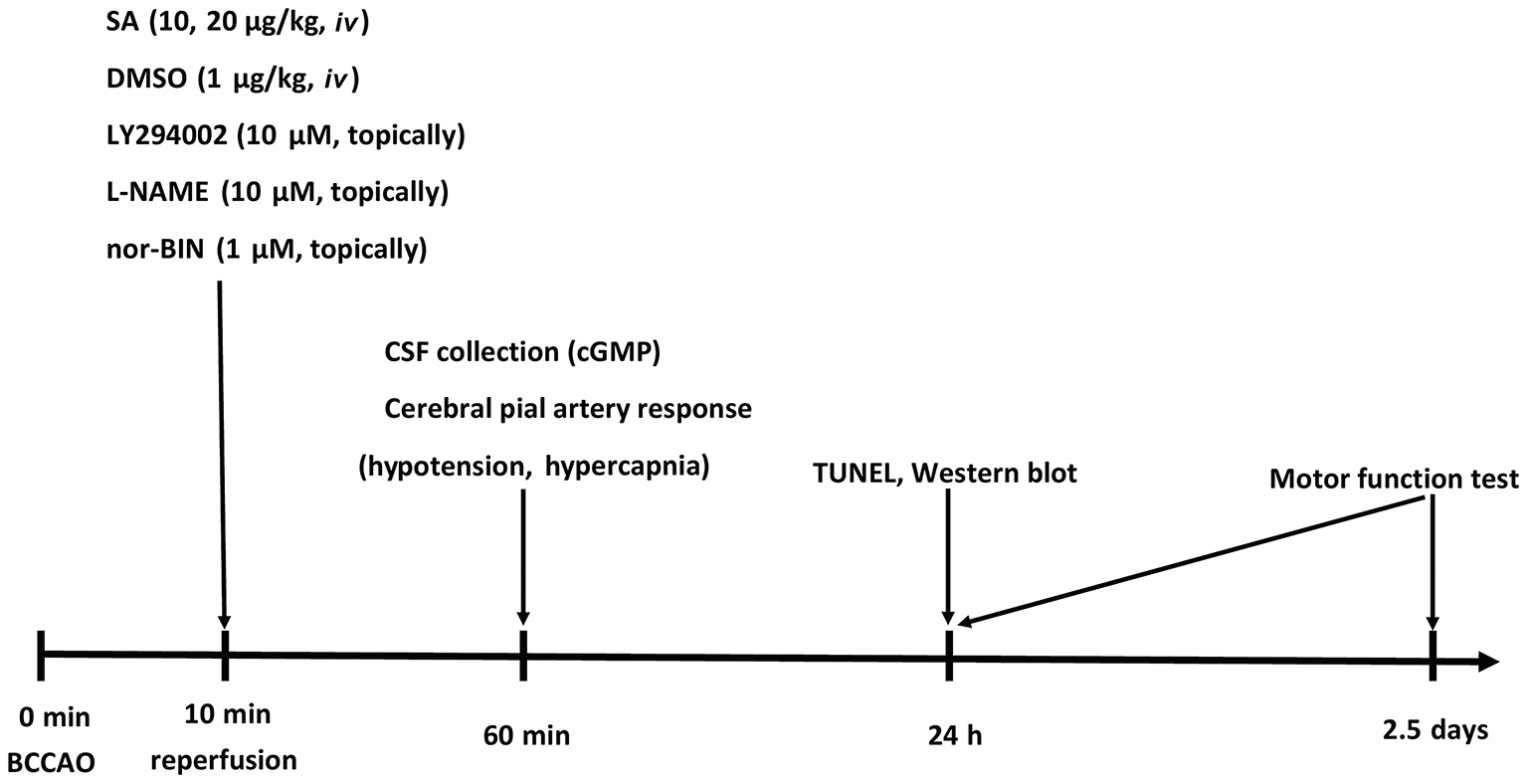
Protocol flowchart. The bilateral common carotid arteries were colligated for 10 min to establish forebrain ischemia followed by reperfusion. SA: salvinorin A; DMSO: dimethyl sulfoxide; nor-BIN: norbinaltorphimine; BCCAO: bilateral common carotid artery occlusion; CSF: cerebrospinal fluid.

**Figure 2.**
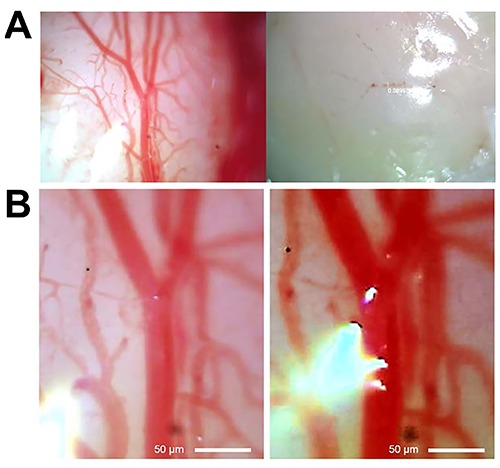
Microscopy of brain cortex after ischemia. *A*, the left panel shows good cerebral perfusion before ischemia, whereas the right panel exhibits a pale appearance after ischemia-induced blood flow blocking, indicating poor perfusion. *B*, the left panel shows normal blood vessel of the cerebral cortex, whereas the right panel exhibits blood vessel dilation, indicating the presence of hypercapnia and hypotension. Scale bar=50 μm.

Thirty Sprague Dawley rats (250–300 g), provided by the Experimental Animal Center of Shanghai Jiaotong University, were used. All animals were acclimatized for 7 days in humidity-controlled housing with natural illumination and allowed to eat and drink freely at room temperature (23±2°C). The protocol flowchart is shown in Figure 1. Bilateral common carotid artery occlusion (BCCAO) was performed to establish the forebrain ischemia model as previously described (17). Rats were intraperitoneally (*ip*) injected with 3% pentobarbital (2 mL/kg) for maintenance of anesthesia. After tracheotomy, bilateral common carotid arteries were isolated and bilateral femoral arteries were catheterized to monitor the blood pressure, blood gas tensions, and pH. The femoral vein was catheterized for medication administration. Heparin (50 U) was given intravenously. Forebrain ischemia was induced by colligation of the bilateral common carotid arteries for 10 min after drawing 6–10 mL arterial blood instead of venous blood to maintain the arterial blood pressure at 25–30 mmHg and guarantee shutdown of pial artery blood flow and the withdrawal of blood that refused to go back to the femoral vein as previously described (11,18). Blood flow blocking was observed, and a pale appearance was exhibited under microscopy after ischemia, indicating a poor perfusion (Figure 2A). Clips were removed after 10 min, and the blood was reinjected. We infused some Lactated Ringer's solution (1∼2 mL) and NaHCO_3_ according to blood pressure and arterial blood gas. The ports attached to the cranial window ring fit 17-gauge hypodermic needles for CSF sampling.

### Drug treatment

Sham-operated animals underwent a similar procedure, with the exception of arterial occlusion. The ischemia reperfusion (IR) control group received DMSO (Amresco, USA, 1 µL/kg, *iv*) administration immediately after ischemia (n=6). Two different doses of salvinorin A (Sigma, USA, 10 and 20 µg/kg, *iv*) were administered with the onset of reperfusion (n=6). The 5th, 6th, and 7th groups received salvinorin A (20 µg/kg, *iv*) and LY294002 (Cayman Chemical, USA, inhibitor of PI3K, 10 µM), L-NAME (Cayman Chemical, inhibitor of NOS, 10 μM) or norbinaltorphimine (norBIN, Sigma, antagonist of KOR, 1 μM), injected topically through cranial window, after ischemia (n=6) as described in previous studies (19–21).

### Cerebral pial artery responses

Cerebral pial artery responses to hypercapnia and hypotension were obtained before ischemia and 1 h after ischemia as previously described (10). Hypercapnia (PaCO_2_ 70 to 80 mmHg) was produced by inhalation of a high concentration of CO_2_mixture gas (10% CO_2_; 21% O_2_; 69% N_2_). Hypotension was produced by the rapid withdrawal of 10∼15 mL/kg of blood from the femoral artery reduced by 45%. A closed cranial window, consisting of a steel ring with a glass cover slip connecting to 3 ports was placed for direct cerebral pial artery visualization and diameter measurement. The normal blood vessel of the cerebral cortex and the blood vessel dilation were visualized on a monitor connected to the microscope (model A60H, Leica Microsystems Inc., Germany) and measured via a video microscale (model VPA 550, For-A-Corp., USA) (Figure 2B). The ports attached to the cranial window ring fit 17-gauge hypodermic needles for CSF sampling, washout, and drug administration. CSF was collected at baseline and 1 h after ischemia for cGMP level measurement. The cerebral cortex after 24 h of ischemia was collected for TUNEL staining and p-AKT detection.

### cGMP levels measurement

Fresh CSF samples were collected as described above. The levels of cGMP were measured by ELISA kits according to the manufacturer's instructions (Sigma-Aldrich, USA).

### Histological study

Twenty-four hours after forebrain ischemia, rats were anesthetized with 3% pentobarbital and intracardially perfused first with 250 mL saline and then with 250 mL 4% paraformaldehyde. Samples were processed by embedding in paraffin blocks, and 5-μm thick sections were stained using HE. Hippocampal damage was determined by calculating the number of injured neurons in the hippocampus, cortex and striatum at a magnification of 400×. The percentage of injured neurons was determined by dividing the number of injured neurons by the total number of neurons in five separate fields.

### TUNEL staining

After 24 h of ischemia, the rats were anesthetized and perfused transcardially with 0.9% saline followed by icy 4% paraformaldehyde solution and fixed for 24–48 h for paraffin embedding. TUNEL staining was performed according to the manufacturer's protocol, and slices were labeled with streptavidin-horseradish peroxidase and TUNEL-positive cells emitted a green fluorescent color. The negative control was labeled without streptavidin-horseradish peroxidase. TUNEL-positive cells were quantified using light microscopy at 400× magnification, and 5 fields for each section were selected from the ischemic adjacent cortex. The average percentage of TUNEL-positive cells was determined using Image-Pro Plus 6.0 image analysis software (Media Cybernetics, USA) and reported through a scale calibration.

### Western blot

Twenty-four hours after forebrain ischemia, the hippocampus, cortex and striatum were obtained and homogenized. Then, protein samples were collected, and the concentration was determined. Proteins (40 µg/lane) were separated and resolved by SDS-PAGE on a 12%-acrylamide gel and transferred to a polyvinylidene fluoride membrane (PVDF, Millipore, USA). The membrane was blocked with 5% skim milk (BD Bioscience, USA) for 1 h at room temperature and incubated with primary antibodies against phosphor-AKT (p-AKT, Thr308) and total AKT (Cell Signaling Technology, USA) overnight. After washing with TBS-T three times, the membranes were incubated with HRP-conjugated secondary antibody (1:2000, Jackson ImmunoResearch Laboratories, Inc., USA) for 1 h at room temperature. Beta-actin (Cell Signaling Technology) was used as an internal control. A Calibrated Densitometer (Bio-Rad, USA) was used to scan immunoblotting images.

### Motor function test

On the 1st, 2nd, and 5th post-ischemia day, rats were subjected to neuromotor tests (screen clinging, horizontal bar, and prehensile traction; 9=best possible score) as previously described (22). The animal was placed on the screen (29×30 cm) when horizontal, and the screen was then rotated into the vertical plane. The time that the rat held on to the vertical screen was recorded to a maximum of 15 s (screen clinging). Next, the animal was placed at the center of a horizontal wooden rod (2.5 cm in diameter), and the time that the rat was able to remain balanced on the rod was recorded for a maximum of 30 s (horizontal bar). Finally, the time that the animal was able to cling to a horizontal rope was recorded to a maximum of 5 s (prehensile traction). The tests were performed by a researcher who was blind to animal group assignment.

### Statistical analysis

All statistical analyses were performed with GraphPad Prism 5.0 software (USA). Data are reported as means±SD. One-way or two-way ANOVA was employed for multiple group comparison. Motor function tests were analyzed using Tukey's multiple comparison test. A P value less than 0.05 was considered statistically significant.

## Results

### Blood pressure, gas tension, and PH

The data of blood pressure, gas tension, and pH are shown in Table 1. At each time point (a, before ischemia; b, onset reperfusion; and c, 1 h after ischemia), there was no significant difference when all groups were compared with each other.

**Table 1.** Blood pressure, gas tension, and pH before ischemia reperfusion (IR), IR onset, and 1 h after IR.

	Sham	IR (DMSO)	SA10	SA20	SA20+LY	SA20+LN	SA20+nor-BIN
MBP							
Before IR	66±6.78	65.4±4.67	63.2±4.72	63.6±4.88	64.2±4.76	65.4±6.43	64±6.12
IR onset	56±6.09	54.2±3.03	54.8±3.03	56.8±6.3	64.6±5.5	53±6.44	54.2±5.62
1 h after IR	64.8±4.97	63.6±6.61	62.2±5.76	66.4±7.6	63.6±3.43	64.4±7.92	60.5±4.85
PO_2_							
Before IR	209.6±11.28	210.8±21.79	232.6±14.58	221.8±9.03	219.6±15.03	218.4±10.57	216.8±21.7
IR onset	125±13.71	113.6±9.78	138.8±18.29	132.2±19.21	115.8±13.26	117±35.7	137.4±35.6
1 h after IR	210±8.91	217±18.48	226±14.84	215±13.95	197.4±20.18	186.6±14.93	201.6±611.23
PCO_2_							
Before IR	41.6±2.61	39.8±1.3	42±5	42.2±2.38	44±2	39.2±2.38	41.6±3.21
IR onset	44.28±3.91	45.6±2.3	43±2.12	38.4±1.15	42.8±3.8	41.8±3.03	42.2±4.43
1 h after IR	41.2±3.96	41.2±5.4	41.4±4.03	44±3.32	43.4±2.89	40.8±3.34	42.8±2.77
pH							
Before IR	7.4±0.039	7.388±0.02	7.421±0.026	7.398±0.039	7.385±0.031	7.402±0.036	7.408±0.025
IR onset	7.29±0.018	7.32±0.038	7.314±0.005	7.311±0.015	7.301±0.015	7.314±0.026	7.308±0.017
1 h after IR	7.397±0.023	7.39±0.04	7.389±0.028	7.38±0.022	7.381±0.026	7.397±0.02	7.366±0.013

Data are reported as means±SD. Data were analyzed by one-way ANOVA followed by Tukey's test. No statistical difference was found among the different groups. LY: LY294002 (10 μM, injected); LN: L-NAME (10 μM, injected topically); IR (ischemia reperfusion control, DMSO): 1 μL/kg (*iv*); SA10: salvinorin A (10 μg/kg, *iv*); SA20: salvinorin A (20 μg/kg, *iv*); norBIN: norbinaltorphimine 1 μM (injected topically).

### Salvinorin A preserved cerebral pial artery autoregulation after cerebral cortex ischemia

**Figure 3.**
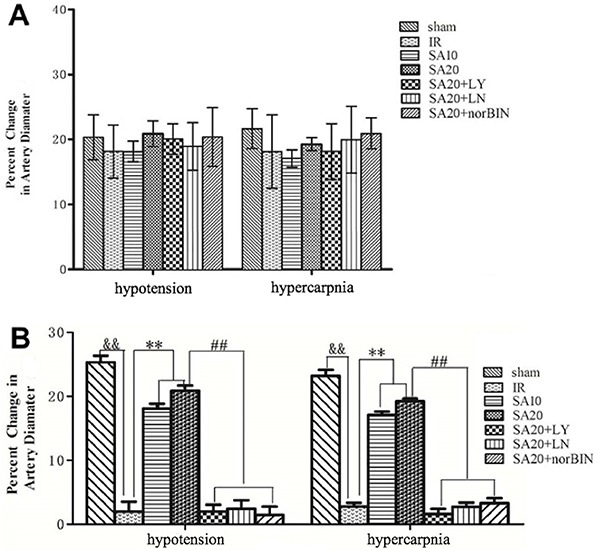
Salvinorin A (SA) preserved cerebral pial artery autoregulation after cerebral cortex ischemia. SA10, SA20, SA20+LY294002, SA20+L-NAME, SA20+norBIN were administered, and the cerebral pial artery autoregulation was evaluated. *A*, No significant differences were found in the pial artery autoregulation among different groups before ischemia. *B*, Pial artery autoregulation among different groups after ischemia. Data are reported as means±SD for n=6. ^&&^P<0.01 compared to the sham control group, **P<0.01 compared to the IR (DMSO) group, ^# #^P<0.01 compared to the SA2 treatment group. The pial artery autoregulation was analyzed by one-way ANOVA followed by Tukey's test. LY: LY294002 (10 μM, injected topically); LN: L-NAME (10 μM, injected topically); IR (ischemia reperfusion control, DMSO): 1 μL/kg (*iv*); SA10: salvinorin A (10 μg/kg, *iv*); SA20: salvinorin A (20 μg/kg, *iv*); norBIN: norbinaltorphimine 1 μM (injected topically).

All animals were monitored with blood gas and temperature and no significant difference was found on the baseline value (results not shown). No significant difference was found in pial artery autoregulation among the different groups before ischemia (Figure 3A). However, the dilation responses of the pial artery to hypercapnia or hypotension were impaired after ischemia and were preserved with different doses of salvinorin A treatment. The preservation of autoregulation was abolished when LY294002 or L-NAME or norBIN was administered, as seen in Figure 3B.

### Salvinorin A increased cGMP levels after cerebral cortex ischemia

**Figure 4.**
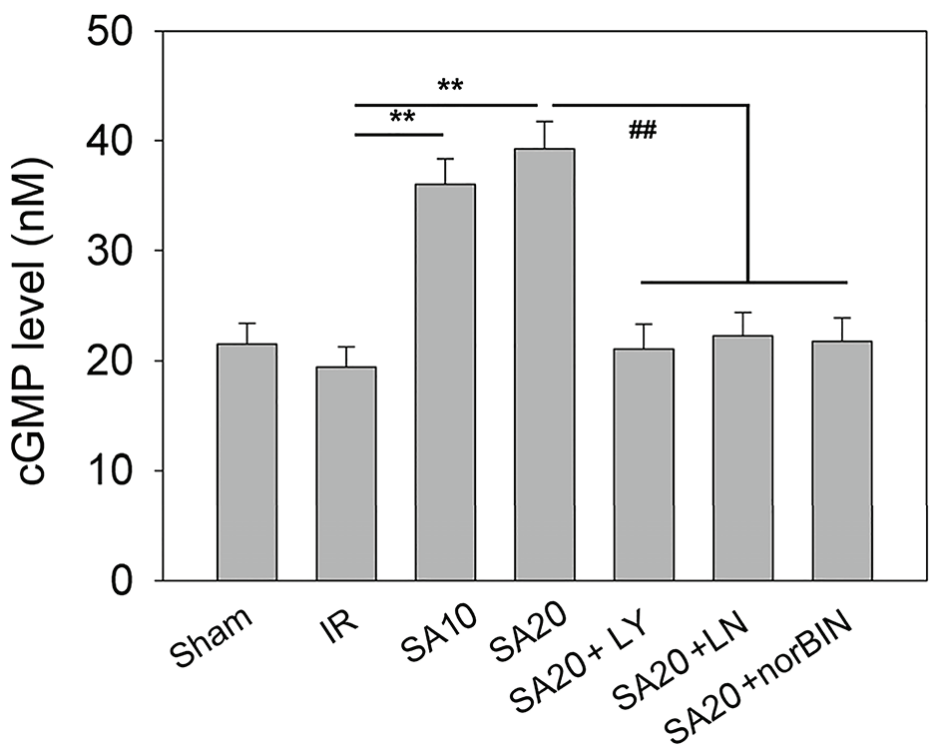
Measurement of cGMP levels in cerebrospinal fluid from different treatment groups after cerebral cortex ischemia. SA10, SA20, SA20+LY294002, SA20+L-NAME, SA20+norBIN were administered and the levels of cGMP were measured. Data are reported as means±SD. **P<0.01 compared to the IR (DMSO) group, ^# #^P<0.01 compared to the SA20 treatment group. The cGMP levels were analyzed by one-way ANOVA followed by Tukey's test. LY: LY294002 (10 μM, injected topically); LN: L-NAME (10 μM, injected topically); IR (ischemia reperfusion control, DMSO): 1 μL/kg (*iv*); SA10: salvinorin A (10 μg/kg, *iv*); SA20: salvinorin A (20 μg/kg, *iv*); norBIN: norbinaltorphimine 1 μM (injected topically).

As shown in Figure 4, cGMP levels did not change after ischemia compared to those after sham control (19.425±1.904 *vs* 21.500±1.894 nM). However, compared to IR (DMSO) control, salvinorin A administration remarkably increased cGMP level (36.050±2.342, 39.250±2.500 *vs* 19.425±1.904 nM). LY294002, L-NAME, or norBIN administration abolished salvinorin A-increased cGMP levels (21.050±2.253, 22.250±2.130, 21.750±2.121 *vs* 39.250±2.500 nM).

### Salvinorin A protected against ischemia-induced neuronal death

**Figure 5.**
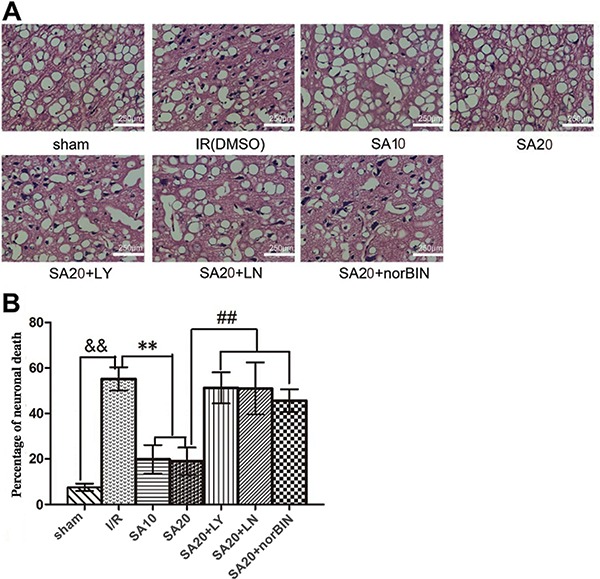
Salvinorin A suppressed ischemia-induced neuronal death, determined by dividing the number of injured neurons by the total number of neurons. Representative histological photographs are shown in (*A*) and data in (*B*). Data are reported as means±SD (n=6). ^&&^P<0.01 compared to the sham control group, **P<0.01 compared to the IR (DMSO) group, ^# #^P<0.01 compared to the SA20 treatment group. Scale bar, 250 μm. The necrotic neurons were analyzed by one-way ANOVA followed by Tukey's test. LY: LY294002 (10 μM, injected topically); LN: L-NAME (10 μM, injected topically); IR (ischemia reperfusion control, DMSO): 1 μL/kg (v); SA10: salvinorin A (10 μg/kg, *iv*); SA20: salvinorin A (20 μg/kg, *iv*); norBIN: norbinaltorphimine 1 μM (injected topically).

Compared to IR (DMSO) control, salvinorin A significantly suppressed ischemia-induced neuronal death, while LY294002 or L-NAME or norBIN could abolish the protective effect of salvinorin A against neuronal death induced by ischemia (Figure 5A and B).

### Salvinorin A protected against ischemia-induced cerebral cortex cell apoptosis

**Figure 6.**
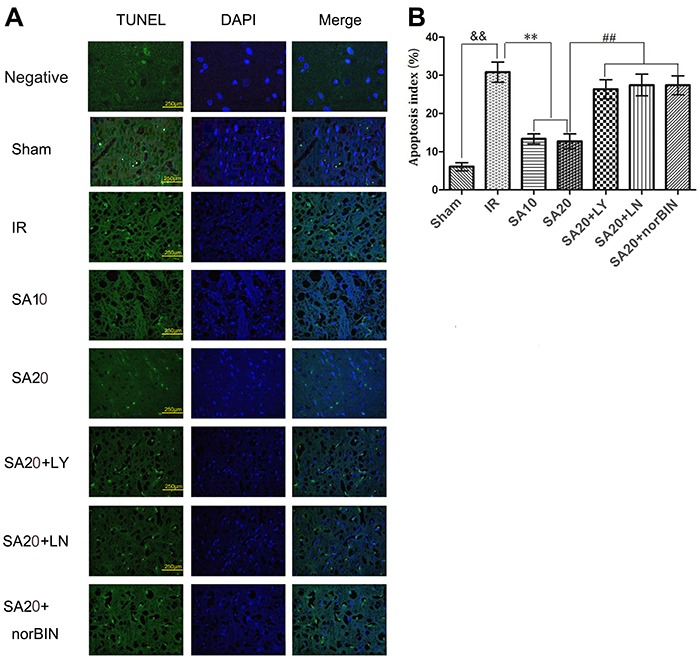
Determination of the cell apoptosis in the cerebral cortex by TUNEL staining. *A*, Representative TUNEL staining images among different treatment groups showed obvious cell apoptosis and increased numbers of apoptotic positive cells after cerebral cortex ischemia for 10 min. Scale bar, 250 μm. The negative control group was without the streptavidin-horseradish peroxidase. *B*, Quantitative analysis of cell apoptosis. Data are reported as means±SD for n=6. ^&&^P<0.01 compared to the sham control group. **P<0.01 compared to the IR (DMSO) group, ^# #^P<0.01 compared to the salvinorin A2 treatment group. Cell apoptosis was analyzed by one-way ANOVA followed by Tukey's test. LY: LY294002 (10 μM, topically injected); LN: L-NAME (10 μM, injected topically); IR (ischemia reperfusion control, DMSO): 1 μL/kg (*iv*); SA10: salvinorin A (10 μg/kg, *iv*); SA20: salvinorin A (20 μg/kg, *iv*); norBIN: norbinaltorphimine 1 μM (injected topically).

A cell apoptosis assay was performed to evaluate the protective effect of salvinorin A on the cerebral cortex after ischemia. Our results showed that compared to IR (DMSO) control, salvinorin A administration significantly suppressed the cerebral cortex cell apoptosis, while LY294002, L-NAME or norBIN abolished the salvinorin A-suppressed apoptosis effect (Figure 6A and B).

### Salvinorin A increased the expression of p-AKT after cerebral cortex ischemia

**Figure 7.**
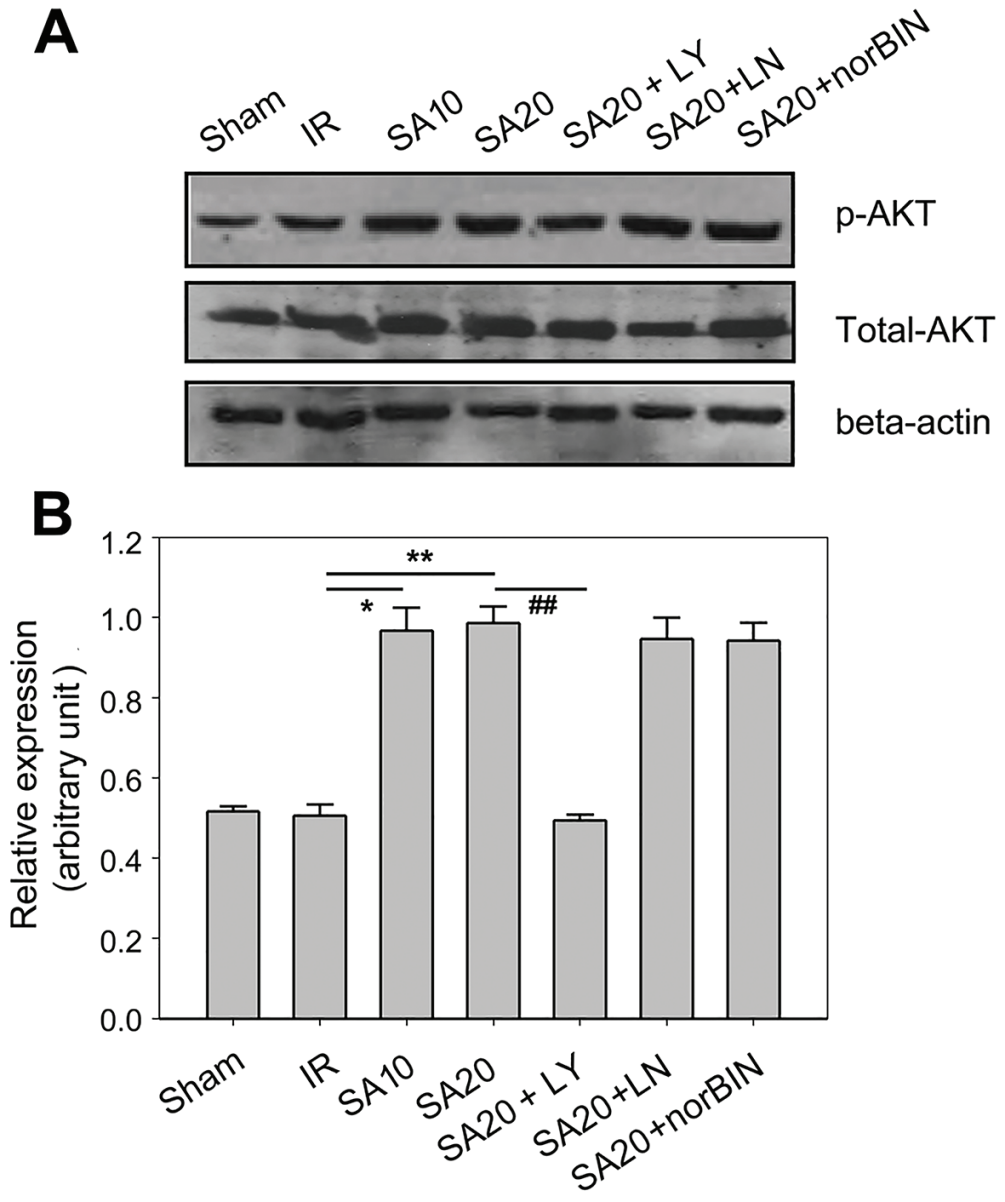
Determination of the expression of p-AKT in different treatment groups after cerebral cortex ischemia. SA10, SA20, SA20+LY294002, SA20+L-NAME, SA20+norBIN were administered and western blot assay was performed to detect the expression of p-AKT. *A*, Representative immunoblotting images among different treatment groups are shown. *B*, Absorbance analysis of the expression of p-AKT. Data are reported as means±SD. *P<0.05, **P<0.01 compared to the IR (DMSO) group, ^# #^P<0.01 compared to the salvinorin A2 treatment group. The expression of p-AKT was analyzed by one-way ANOVA followed by Tukey's test. LY: LY294002 (10 μM, injected topically); LN: L-NAME (10 μM, injected topically); IR (ischemia reperfusion control, DMSO): 1 μL/kg (*iv*); SA10: salvinorin A (10 μg/kg, *iv*); SA20: salvinorin A (20 μg/kg, *iv*); norBIN: norbinaltorphimine 1 μM (injected topically).

The molecular mechanism involved in the cerebral cortex after ischemia was also explored. p-AKT levels did not change after ischemia compared to those after the sham control. However, salvinorin A administration remarkably increased p-AKT level (P<0.05, SA10 compared to IR; P<0.01, SA20 compared to IR), whereas LY294002 abolished salvinorin A-increased p-AKT levels (Figure 7A and B).

### Salvinorin A improved the motor function score after cerebral cortex ischemia

**Figure 8.**
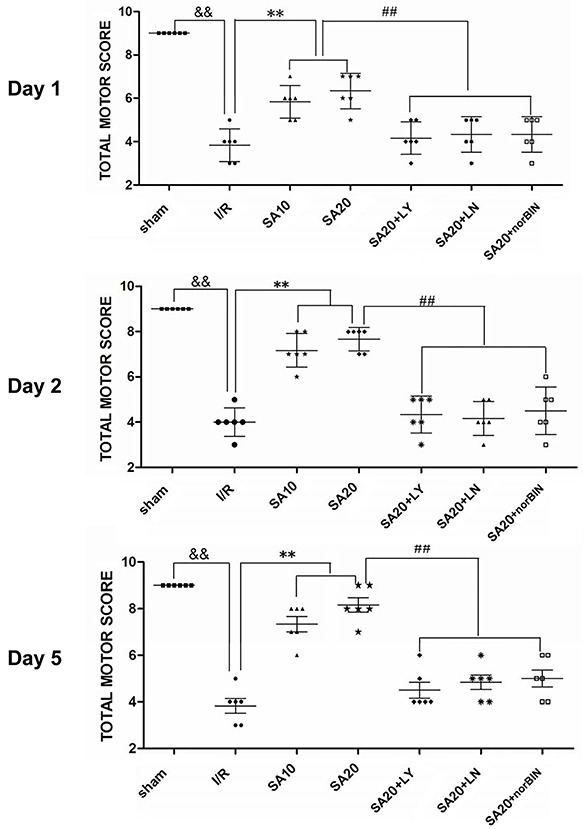
Effect of salvinorin A treatment on motor function was evaluated on days 1, 2, and 5 after forebrain ischemia. SA10, SA20, SA20+LY294002, SA20+L-NAME, SA20+norBIN were administered and the motor function of rats with forebrain ischemia was evaluated. Horizontal bars represent the group median values and symbols represent values for individual rats in each treatment group. ^&&^P<0.01 compared to the sham control group, **P<0.01 compared to the IR (ischemia reperfusion control, DMSO) group, ^# #^P<0.01 compared to the salvinorin A20 treatment group. Motor function tests were analyzed using Tukey's multiple comparison test. LY: LY294002 (10 μM, injected topically); LN: L-NAME (10 μM, injected topically); IR (DMSO): 1 μL/kg (*iv*); SA10: salvinorin A (10 μg/kg, *iv*); SA20: salvinorin A (20 μg/kg, *iv*); norBIN: norbinaltorphimine 1 μM (injected topically).

Motor function test (TMS) score system was used to evaluate the motor function on days 1, 2, and 5 after ischemia induction. Compared to IR (DMSO) control, salvinorin A administration significantly improved the motor function of the rats on days 1, 2, and 5. However, LY294002, L-NAME, or norBIN administration abolished salvinorin A-improved motor function of the rats on days 1, 2, and 5 (Figure 8).

## Discussion

In our study, BCCAO was used as a model of transient global cerebral ischemia according to previous reports. The BCCAO model simulates the effects produced by the systemic decrease in blood flow, affects the entire brain, and clinically resembles transient global cerebral ischemia in a number of pathological aspects such as cardiac arrest, drowning or systemic hypotension during surgery. Global cerebral ischemia leads to neurological deficits in memory and executive function (23). Injury of a brain vessel, which could further increase the severity of cerebral ischemia, is the most common phenomenon during the cerebral ischemia (24). Early prevention of cerebral vessel dysfunction and improvement of the cerebral damage is the key step in neuronal protection.

In the present study, we investigated the role of salvinorin A in cerebral pial artery protection in a rat model of forebrain ischemia injury. We found no significant difference in the pial artery autoregulation among different groups before ischemia. However, the dilation responses of the pial artery to hypercapnia or hypotension were impaired after ischemia. Hypercapnia was produced by inhalation of high concentration CO_2_mixture gas. Hypotension produced by the rapid withdrawal of 10∼15 mL/kg of blood from the femoral artery was reduced by 45%. Two different levels of treatments were used in the pre-experiments, and we found that the results of treatment with low levels of hypercapnia and hypotension were not ideal. Thus, we eventually gave up on the experimental results of low level processing in our final research. We found that two doses of salvinorin A treatment preserved the autoregulation of cerebral pial artery. Salvinorin A treatment protected the brain tissues from ischemia injury, which is demonstrated by decreased cell apoptosis around the ischemia area and recovery of motor function at 1 to 5 days.

Autoregulation function during cerebral ischemia is the main physiological activity to maintain normal brain metabolism. Loss of autoregulation could result in disruption of brain metabolism, induction of brain edema, and increase the severity (25), which were consistent with our results on abnormal pial artery dilation, increasing cell apoptosis and severe motor function injury. The previous study has shown that salvinorin A treatment exerted vessel dilation function in normal brain tissue (9). In our study, we applied the salvinorin A immediately after ischemia, and it still exerted protective effects and maintained the normal response to cerebral pial artery autoregulation (10,20). We also observed the effect of different doses of salvinorin A on cerebral pial artery response, and the results showed that both doses were effective.

The PI3K/AKT pathway is considered a critical signaling pathway in multiple diseases. AKT phosphorylation is an important part of this pathway. Yoshitomi et al. demonstrated that phosphorylation of eNOS and Akt was decreased in the cerebral cortex of stroke-prone spontaneously hypertensive rats compared with that in Wistar-Kyoto rats (26). After reperfusion, increased levels of p-AKT and p-eNOS were observed transiently from 0.5 to 2 h and from 4 to 12 h, respectively, whereas both of them were much lower at 24 h after reperfusion than the sham group (27,28). The regulatory effect of salvinorin A on the AKT phosphorylation is poorly understood. Therefore, we investigated the effect of salvinorin A on the phosphorylation level of AKT. Our results showed that salvinorin A significantly increased the expression of AKT phosphorylation. The PI3K inhibitor LY294002 abolished the salvinorin A-increased AKT phosphorylation rather than NOS antagonist L-NAME and KOR antagonist norBIN, suggesting that salvinorin A may regulate the PI3K/AKT pathway through other ways. Previous studies have shown that salvinorin A preserved cerebrovascular autoregulation to hypotension and hypercapnia after brain hypoxia/ischemia via ERK/MAPK in a piglet model (9,10). Recently, we proposed that salvinorin A might also regulate PI3K/AKT pathway through activating platelet release. We are currently conducting further studies to confirm this.

NO is the key factor that regulates vessel tension, while NOS is the rate-limiting enzyme in NO generation. Diffusion of NO from vessel wall to the vascular smooth muscle cells could activate cGMP and result in the relaxation of smooth muscle cells that eventually leads to the dilation of the vessel (29). PI3K/AKT/eNOS was considered the key step for NO generation during vessel dilation (30). Previous studies have shown that increased eNOS level could result in vessel dilation, improvement of endothelial dysfunction (31) and decrease brain injury during ischemia (32,33). In the present study, the level of cGMP in CSF was attenuated 1 h after ischemia, although no significant difference was detected between the IR group and sham control group. Our results also showed that salvinorin A treatment elevated the levels of cGMP in CSF. The PI3K inhibitor LY294002, NOS antagonist L-NAME and KOR antagonist norBIN could abolish salvinorin A-increased levels of cGMP, suggesting that the PI3K/AKT/cGMP pathway was involved in the regulatory process of salvinorin A on cGMP. However, the detail mechanisms involved need to be further explored.

KOR has been demonstrated to protect the brain against cerebral ischemia injury. We found here that salvinorin A administration at a dose of 10 or 20 µg/kg after ischemia preserved autoregulation of the cerebral pial artery to hypercapnia and hypotension via the PI3K/AKT/cGMP pathway. Multiple mechanisms have been proposed to be involved in the regulatory role of KOR. For example, BRL52537 protects against cerebral IR injury via a mechanism involving STAT3 signaling (34); U-50488H exerts its effect via regulation of Na(+), K(+) ATPase activity (35). Induction of NO could also be generated by KOR agonists (36). A previous study has shown that KOR agonist could decrease the cell apoptosis in the setting of myocardial ischemia through PI3K-dependent AKT phosphorylation, while NO could be generated by NOS (eNOS) phosphorylation during the same process (37). Although KOR agonists exhibit tremendous therapeutic value, most KOR agonists have not been applied in clinical settings because of their intrinsic characteristics as opioids (low selectivity and/or lack of an acceptable safety profile). However, the detailed mechanisms of salvinorin A on the dilation function of brain vessels have not been fully explored. Further studies are needed to elucidate the upstream and downstream mechanism for salvinorin A modulation of the PI3K/AKT pathway in brain tissues. Based on our present results, we proposed that salvinorin A may alleviate inflammation and edema induced by cerebral ischemia.

There are some limitations in this study. Firstly, only two doses of salvinorin A were used in the treatment of ischemia; however, we are not sure that an increased dose would result in adverse reactions. Secondly, the application of salvinorin A at other time points after ischemia should also be tested to find the optimal choice.

In conclusion, our findings have shown that salvinorin A could preserve cerebrovascular autoregulation in response to hypotension and hypercapnia after forebrain ischemia via the PI3K/AKT/cGMP pathway, which could result in decreasing of neuronal death and improvement of motor function.
